# Variability extraction and modeling for product variants

**DOI:** 10.1007/s10270-015-0512-y

**Published:** 2016-01-29

**Authors:** Lukas Linsbauer, Roberto Erick Lopez-Herrejon, Alexander Egyed

**Affiliations:** Institute for Software Systems Engineering, Johannes Kepler University, Linz, Austria

**Keywords:** Feature, Trace, Product variant, Variability, Dependency

## Abstract

Fast-changing hardware and software technologies in addition to larger and more specialized customer bases demand software tailored to meet very diverse requirements. Software development approaches that aim at capturing this diversity on a single consolidated platform often require large upfront investments, e.g., time or budget. Alternatively, companies resort to developing one variant of a software product at a time by reusing as much as possible from already-existing product variants. However, identifying and extracting the parts to reuse is an error-prone and inefficient task compounded by the typically large number of product variants. Hence, more disciplined and systematic approaches are needed to cope with the complexity of developing and maintaining sets of product variants. Such approaches require detailed information about the product variants, the features they provide and their relations. In this paper, we present an approach to extract such variability information from product variants. It identifies traces from features and feature interactions to their implementation artifacts, and computes their dependencies. This work can be useful in many scenarios ranging from ad hoc development approaches such as clone-and-own to systematic reuse approaches such as software product lines. We applied our variability extraction approach to six case studies and provide a detailed evaluation. The results show that the extracted variability information is consistent with the variability in our six case study systems given by their variability models and available product variants.

## Introduction

Several technological and economical trends have made it necessary for software products to be readily and efficiently available in different variants that cater to different software platforms, hardware support or customer functionality.

*Variability* is the capacity of software artifacts to vary [37]. Its effective management requires variability information such as the set of possible product variants, the features they provide, how they are related, and how they are implemented. For the latter, we compute traces from features and feature interactions to their implementation artifacts and vice versa.

A *Trace* is a link between a source and a target artifact [10]. Traceability is defined as the potential for traces to be established and used. Variability management is paramount for coping with scenarios where multiple product variants must be developed and maintained such as:

*Supporting and enhancing clone-and-own reuse*. Clone-and-own is a manual ad hoc software reuse approach where new product variants are created by reusing parts from already-existing variants [13]. The parts to be reused must first be located in the existing variants, then extracted, merged, and completed to obtain the new working variant. This process is repeated for each new variant required. This approach is simple, intuitive, and requires only very little upfront investment. However, it inevitably leads to maintenance issues and hinders efficient reuse. For example, bug fixes must be applied to every product variant individually because they do not share a common platform, and identifying reusable implementation is difficult within a large set of product variants. Variability information in this context helps to locate reusable features and their implementing artifacts. It even makes it possible to partially automate reuse and provide more robust support for clone-and-own [15].

*Reverse engineering software product lines (SPLs)*. SPLs are families are families of related systems whose members—variants of a product—are distinguished by the set of features they provide [7, 31]. Ideally these product variants are not maintained individually like in the case of clone-and-own but rather part of a common integrated platform that manages common assets. In cases where the problem domains and the product variants are mature and stable, software companies can consider the development of such a fully integrated SPL platform to reap the documented benefits that SPLs enable such as improved quality, reduced long-term costs, and easier maintenance [31]. In this scenario, variability information is not only highly useful but even necessary for reverse engineering the artifacts needed by SPL development approaches [15, 17, 39].

*Extending an SPL*. In cases where an SPL already exists, the need of providing new features to meet new customer requirements may still arise. In such cases, product lines have to be extended to provide such new features, a process that requires knowledge about already-existing features and how they interrelate. Unfortunately, while every SPL has inherent variability, the information about it is often not explicitly available as many SPLs are the result of ad hoc development. Variability information can be spread out across several places and implemented using various different techniques like preprocessors, configuration files, runtime constructs, and hidden in custom product line configuration tools [24].

In this paper, we present an approach for extracting variability information from sets of related product variants. We work under two basic assumptions:

- The *set of features* provided by each product variant is known (although it is not known where the features are actually implemented in the artifacts). Note that this assumption does *not* require a feature model or any other kind of variability model to be available.
- All *implementation artifacts* for each product variant are available.

We argue that these two requirements are reasonable assumptions for product variants of commercial relevance to companies regardless of how they are maintained or implemented. Obviously the implementations for the product variants need to be available, otherwise, they could not be maintained and sold. The concept of features may sometimes not explicitly be present, but in such cases we found that features can often be retrieved from various sources, e.g., from other departments within an organization (e.g., the sales department must have a concept of features for determining what product variants they can sell), from configuration options in the software (the software of course needs to be able to reflect the feature choices made during the sales process) or by interviewing developers [16].

Our work extracts traces from features as well as feature interactions to their implementation artifacts and computes trace dependencies. This variability information is modeled and represented in a way that can be beneficial for development scenarios such as those described above. Our previous work also computed traces [25], but could not deal with artifacts that had a non-unique trace, ordered artifacts (e.g., statements in a programming language) and instead of organizing artifacts in a tree structure it employed a simple list. Our follow-up work improved on these aspects [15] but still did not consider dependencies between artifacts contained in different traces and their consistency with domain knowledge available for example in the form of variability models. The evaluation was solely based on the implementation of product variants. We therefore extend our previous work (see Linsbauer et al. [15, 25]) by:

- Extending the extraction process to also extract dependencies between traces and depicting them as dependency graphs.
- Evaluating extracted traces and dependency graphs with respect to the feature model (as a form of variability model) of case studies when available.
- Presenting a more detailed evaluation and analysis of the extraction process, based on specialized metrics such as number of extracted traces or runtime per product variant used as input. This is to provide an empirical gist of how variability is implemented in practice and how and why this approach is applicable and useful and to reveal where potential optimizations can be made.

Replication material can be found on the website of the Institute for Software Systems Engineering at the Johannes Kepler University Linz: http://www.jku.at/isse/content/e139529/e126342/e219248/e289826.

The remainder of this paper is structured as follows: Sect. 2 introduces a running example and basic background. Section 3 discusses a motivating scenario and highlights the challenges that need to be addressed. Section 4 describes data structures and operations on them which will be used in Sect. 5 to explain the trace extraction algorithm for tracing features and their interactions to implementation artifacts. Section 6 describes the extraction of dependencies between feature traces. Section 7 provides a detailed evaluation and analysis of the presented approach on six case studies from different domains and of varying size. Finally we present related work in Sect. 8 and conclude with a summary in Sect. 9 and an outlook on future work in Sect. 10.

## Background and running example

In this section, we introduce our running example, a set of simple software variants, to illustrate the challenges faced for extracting variability information. In addition, we provide the basic terminology of SPLs and of our approach.

### Running example and basic definitions

**Fig. 1:**
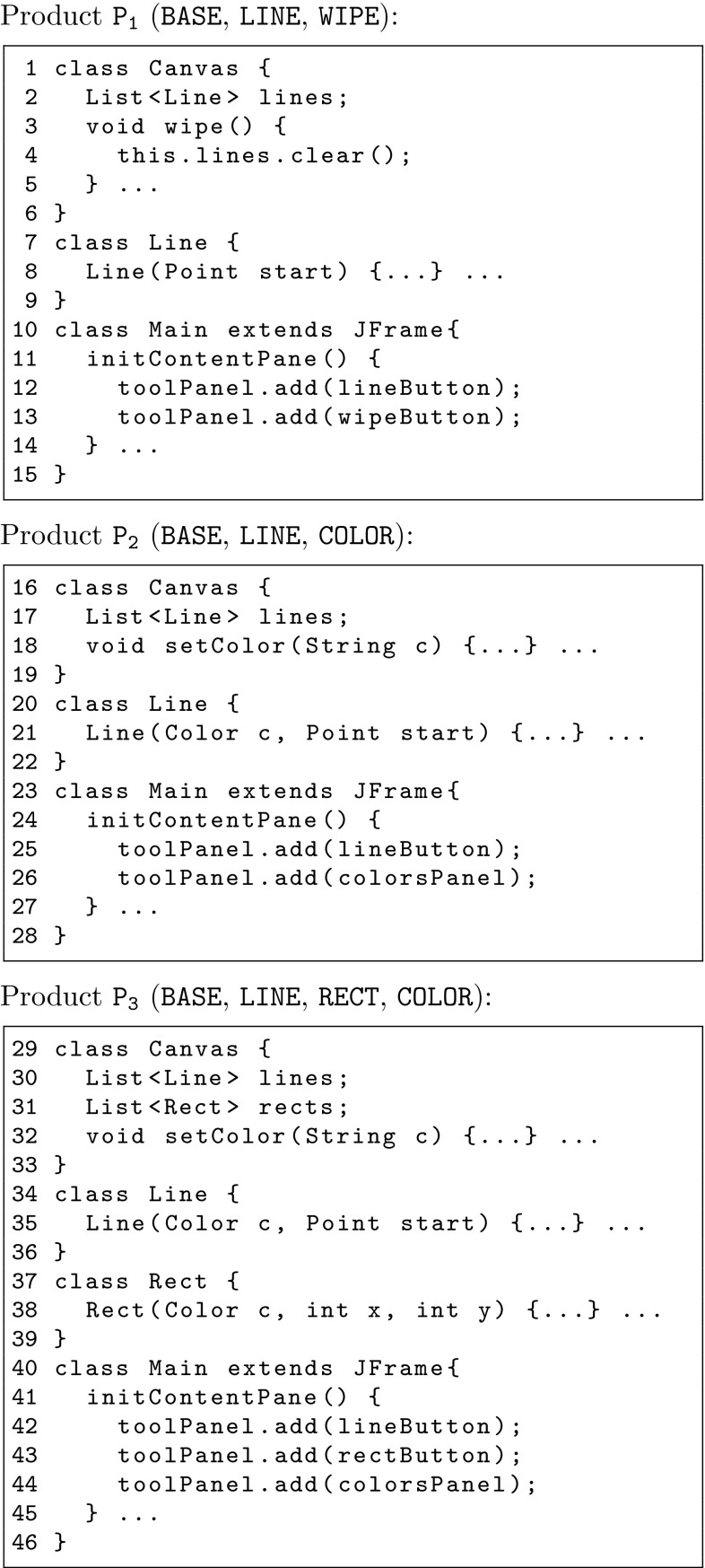
Source code snippets for drawing application product variants

The starting point of our work is a set of existing product variants, and for each variant we require the knowledge of what features it provides. Let us consider a set of simple drawing applications as our running example. Each variant supports a subset of the following features: the ability to handle a drawing area ($\mathtt {{{base}}}$), draw lines ($\mathtt {{{Line}}}$) and rectangles ($\mathtt {{{Rect}}}$), select a color to draw with ($\mathtt {{{Color}}}$), and wipe the drawing area clean ($\mathtt {{{Wipe}}}$). Let us assume that three variants $\mathtt {{P_1}}$, $\mathtt {{P_2}}$, and $\mathtt {{P_3}}$ are available already, each providing a distinct set of features, see Table 1, and each having its own distinct implementation, see code snippets in Fig. 1.

**Table 1:** Initial drawing application product variants

Products	$\mathtt {{{Base}}}$	$\mathtt {{{Line}}}$	$\mathtt {{{Rect}}}$	$\mathtt {{{Color}}}$	$\mathtt {{{Wipe}}}$
Product $\mathtt {{P_1}}$	$\checkmark$	$\checkmark$			$\checkmark$
Product $\mathtt {{P_2}}$	$\checkmark$	$\checkmark$		$\checkmark$	
Product $\mathtt {{P_3}}$	$\checkmark$	$\checkmark$	$\checkmark$	$\checkmark$	

#### **Definition 1**

(*Product Variant*) A *Product Variant* ${\mathtt {{P}}} \in \mathbb {P}$ is a relation $\mathtt {(Features, AT)}$ where $\mathtt {Features} \subseteq \mathbb {F}$ is the set of features that ${\mathtt {{P}}}$ provides and $\mathtt {AT}$ is an artifact tree. An artifact tree is a generic tree structure comprised of a set of implementation artifacts $\mathtt {I} \in \mathbb {I}$ that represents a concrete composition of the artifacts $\mathtt {I}$ that is specific to product variant ${\mathtt {{P}}}$ and implements the features ${\mathtt {{P}}}$ provides. $\mathbb {P}$ denotes the universe of all product variants, $\mathbb {F}$ the universe of all features and $\mathbb {I}$ the universe of all implementation artifacts. We, respectively, denote the $\mathtt {Features}$ and $\mathtt {AT}$ elements of product variant ${\mathtt {{P}}}$ with $\mathtt {P.Features}$ and $\mathtt {P.AT}$.

Artifacts realize the implementation of product variants and can be anything from source code to models, test cases or requirements, etc. The generic tree structure we devised is capable of representing their hierarchy and order. We provide more details in Sect. 4.

A challenge is that the behavior of a single feature may depend on the presence or absence of other features. This fact that features influence each other is referred to as *feature interaction* and is a well-known problem in software reuse [3]. To distinguish whether artifacts of a product implement a single feature or a feature interaction we introduce a notation and terminology inspired by Liu et al. [27]. We use *modules*, a concept more descriptive than features to express relations between features and implementation artifacts. We distinguish *modules* of two kinds: $\mathtt {{{base}}}$ modules and $\mathtt {{{derivative}}}$ modules.

#### **Definition 2**

(*Module*) A *module* is a set of signed (positive or negative) features. A *base module* is a module that consists of *exactly one* positive feature and no negative features. A *derivative module* contains *at least one* positive feature and any number of negative features.

A *base module* ${\mathtt {{{f}}}} = \{ {\mathtt {{{F}}}} \}$ labels artifacts that implement a given feature ${\mathtt {{{F}}}} \in \mathbb {F}$ without any feature interactions. We refer to them with the feature’s name written in lowercase. For example consider field ${{\texttt {{List<Line> lines}}}}$ in Lines 2, 17 and 30 of Fig. 1, for product variants $\mathtt {{P_1}}$, $\mathtt {{P_2}}$ and $\mathtt {{P_3}}$, respectively. This artifact, code in our case, belongs to the base module $\mathtt {{{line}}}$ because it must be present in all product variants that include feature $\mathtt {{{line}}}$ independently of any other features/interactions.

A *derivative module* ${\mathtt {\delta {}^{n}({{\mathtt {{{F}}}}, f_1, ..., f_{n}}})} = \mathtt {\{ {\mathtt {{{F}}}}, f_1, ..., f_{n} \}}$ labels artifacts that implement an interaction between $n+1$ features, where ${\mathtt {{{F}}}} \in \mathbb {F}$ is a positive feature and $f_i$ is ${\mathtt {{{F}}}}_i \in \mathbb {F}$ (if feature ${\mathtt {{{F}}}}_i$ is selected) or $\lnot {}{\mathtt {{{F}}}}_i$ (if not selected), and *n* is the order of the derivative. A derivative module of order *n* thus represents the interaction of $n+1$ features. We treat derivative modules simply as sets of cardinality $n+1$ containing all features (positive or negative) that are involved in the interaction. A derivative module of order $n=0$ is a base module. An example of a derivative module is the constructor for class Line in Fig. 1 which is found in all three variants but with different arguments. The constructor Line(Point start) in Line 8 reflects the situation where feature $\mathtt {{{line}}}$ is selected but feature $\mathtt {{{color}}}$ is not. This artifact corresponds to the derivative module ${\mathtt {{{\delta {}^{1}(Line, \lnot color)}}}}$. Similarly, the constructors in Line 21 and Line 35 have an argument for color, which reflects the fact that features $\mathtt {{{line}}}$ and $\mathtt {{{color}}}$ are selected, and represent the artifacts of the derivative module ${\mathtt {{{\delta {}^{1}(Line, color)}}}}$.

We define a number of auxiliary functions for working with features and modules.

#### **Definition 3**

(*Negate Features* *nF*) To compute for a set of features *F* the set $\bar{F}$ of the same features negated:

$$\begin{aligned} nF(F) = \{ \lnot f \mid f \in F \}. \end{aligned}$$

#### **Definition 4**

(*Compute Modules from Features* *f*2*m*) To compute the set of modules from a set of positive (i.e., selected) features *F* and a set of negative (i.e., not selected) features $\bar{F}$:

$$\begin{aligned} f2m(F, \bar{F}) = \{ p \cup n \mid p \in 2^F \setminus \emptyset \wedge n \in 2^{\bar{F}} \}. \end{aligned}$$

#### **Definition 5**

(*Update Modules with Features* *uM*) To update a set of modules *M* with a set of previously unknown features $\bar{F}$:

$$\begin{aligned} uM(M, \bar{F}) = \{ m \cup n \mid m \in M \wedge n \in 2^{\bar{F}} \}. \end{aligned}$$

### Variability modeling with feature models

Variability models describe what the valid product variants are that form an SPL. *Feature Models (FMs)* are the de facto standard for research on variability modeling that describe the *features*—increments in program functionality [7]—of a software system and their relations [20]. We use feature models to help us depict better the case studies used for evaluation as well as to help us assess the quality of the variability information that our approach extracts. However, we should stress that variability models (feature models or otherwise) are not a requisite or assumption for our approach to be applicable. These points will be further elaborated in the upcoming sections.

**Fig. 2:**
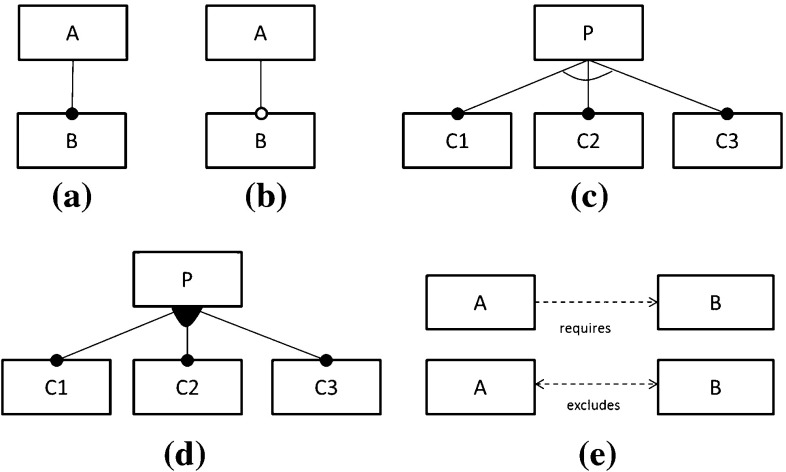
Feature model notation

A feature model is a tree-like structure with the nodes being features. The root node of a feature model is always included in all product variants. A feature can only be part of a product variant if its parent feature is also part of it. A feature can be *mandatory* (denoted with a filled circle at the child end of an edge, see Fig. 2a) or *optional* (denoted with an empty circle at the child end of an edge, see Fig. 2b). A mandatory feature is part of a product variant whenever its parent feature is. An optional feature may or may not be part of a product if its parent is. Features can be grouped into:

- an *inclusive-or* relation, denoted with a filled arc (see Fig. 2d), where one or more features of the group can be selected, or
- an *exclusive-or* relation, denoted with an empty arc (see Fig. 2c), where exactly one feature must be selected.

In addition to parent–child relations, there can also be relationships between features across the tree structure of the feature model. These are called *cross-tree constraints (CTCs)*. A *requires* constraint expresses that the presence of a feature $\mathtt {{{A}}}$ implies the presence of another feature $\mathtt {{{B}}}$ and is denoted as a dashed single-arrow line from $\mathtt {{{A}}}$ to $\mathtt {{{B}}}$ (see top of Fig. 2e). An *excludes* constraint expresses that if a feature $\mathtt {{{A}}}$ is selected another feature $\mathtt {{{B}}}$ must not be selected which is denoted as a dashed double-arrow line between $\mathtt {{{A}}}$ and $\mathtt {{{B}}}$ (see bottom of Fig. 2e). Extra constraints that cannot be expressed by these means are usually added to the feature model in the form of propositional logic expressions [8].

## Motivating scenario

Let us use the case of a clone-and-own scenario to motivate our need for variability information. The starting point of this scenario is a set of existing product variants and for each variant the knowledge of what features it implements. In practice, when developing a new product variant within such context, usually the existing product variant that is most similar to the new one is cloned and then modified and extended, possibly by selectively cloning implementation fragments from other existing product variants [13]. However, this task is mostly carried out manually and performed in a very ad hoc and undisciplined manner, which makes it not only complex but also prone to errors and time-consuming. Already-existing implementation of features and functionality may easily be missed causing their re-implementation, which is not only a waste of time but also has a negative impact on maintainability because the same features are implemented several times in different ways across different product variants.

We envision a more structured and systematic approach for developing a new product variant in a clone-and-own context with the potential to be partially automated. It consists of three steps:

1. *Extract* implementation fragments from existing product variants that will be reused in the new variant.
2. *Compose* the extracted implementation fragments to form the new variant.
3. *Complete* the new variant, if needed, by adding, for example, features and interactions that did not yet exist in any existing variant.

This steps demand detailed knowledge of all product variants and their implementation artifacts. Without this detailed knowledge, for instance, artifacts can be easily missed or misidentified leading to extracted fragments with missing or unnecessary implementation. What makes the extraction task specially difficult is the identification of implementation fragments that are responsible for interactions among features [2]. The manual composition requires the merging of all relevant implementation artifacts while remaining faithful to structure and artifact dependencies. This step is difficult because merged implementation fragments are rarely correct or complete. Finally, the completion step has to fill in missing artifacts that could not be found during the extraction (i.e., new features and feature interactions, or perhaps artifacts that were overlooked by software engineers). The completion also has to address the shortcomings of manual extraction and composition. For example, misidentified artifacts need to be eliminated or repaired. All this additional completion work may lead to different implementations of the same functionality which in turn can make the future maintenance of variants a significantly harder endeavor.

**Fig. 3:**
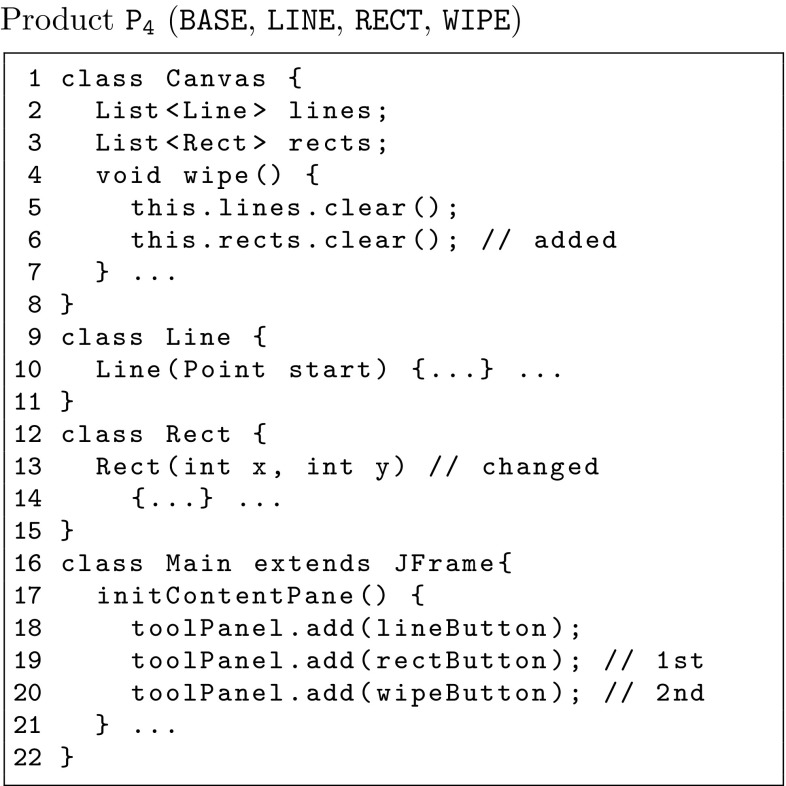
Source code for *Completed* product $\mathtt {{P_4}}$

Now, let us consider that we want to extend the set of drawing applications by creating a new product variant $\mathtt {{P_4}}$ with features $\mathtt {{{Base}}}$, $\mathtt {{{Line}}}$, $\mathtt {{{Rect}}}$ and $\mathtt {{{Wipe}}}$, by applying clone-and-own. The goal is to extract as much code from $\mathtt {{P_1}}$, $\mathtt {{P_2}}$, and $\mathtt {{P_3}}$ as possible. For example, a software engineer might start off by copying the entire product variant $\mathtt {{P_1}}$ into $\mathtt {{P_4}}$ because it is a “close fit” and then extract and compose code for feature $\mathtt {{{Rect}}}$ from product variant $\mathtt {{P_3}}$. Doing so is not trivial. For example, we would need to copy the Rect class from $\mathtt {{P_3}}$ to $\mathtt {{P_4}}$ but change its constructor as it currently has a Color c argument (feature $\mathtt {{{Color}}}$ was not selected for $\mathtt {{P_4}}$). So feature $\mathtt {{{Rect}}}$ without feature $\mathtt {{{Color}}}$ behaves differently, and therefore, the extracted code from $\mathtt {{P_3}}$ contains *surplus code* that the software engineer has to remove. Figure 3 depicts a possible realization of product variant $\mathtt {{P_4}}$.

There are other problems, however. Since feature $\mathtt {{{Rect}}}$ has never appeared with feature $\mathtt {{{Wipe}}}$ before, there is no code that can be associated with module ${\mathtt {{{\delta {}^{1}(Rect, Wipe)}}}}$. Indeed, without this feature interaction the new variant would fail to wipe rectangles. The software engineer would have to add this *missing code* (see Line 6 in Fig. 3). Moreover, the software engineer would also need to decide on the order of certain statements. Consider for instance method initContentPane() in Line 17 of Fig. 3. While it may be clear that the buttons associated for drawing lines and rectangles and for wiping the drawing area clean need to be added to the drawing area, it is not obvious in what order they should be added. Looking at the existing three product variants shows that the button for drawing lines always goes first in this concrete set of drawing applications; however, it is not clear in which order the buttons for rectangles and wiping shall appear as the feature interaction among features $\mathtt {{{rect}}}$ and $\mathtt {{{wipe}}}$ has not been present in any of the three existing product variants. The software engineer then has to decide manually on an order, see for example Lines 19 and 20 in Fig. 3.

**Fig. 4:**
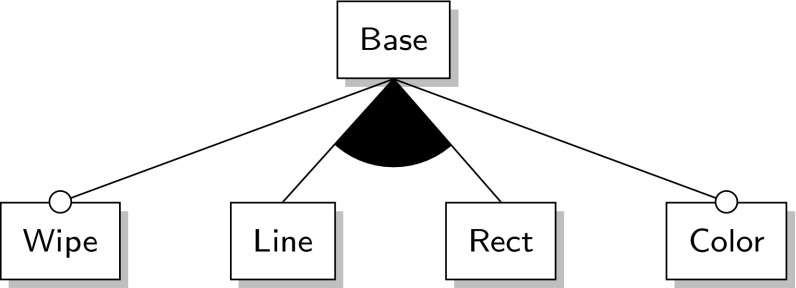
Feature model for the draw case study

Furthermore, let us assume now that we want to create more product variants. This process that we just described for product variant $\mathtt {{P_4}}$ should be repeated anew for each new variant. It should be noted though that once a certain number of product variants is reached, it may pay off to refactor these variants into an SPL instead of dealing with them individually [18]. For our motivation scenario, let us make a product line, *Draw Product Line (DPL)*, out of our draw variants that supports the same five features; a total of twelve different variants shown in Table 2. The feature model of DPL is shown in Fig. 4. There are several techniques to reverse engineer feature models based on the features of their variants, for example refer to [17, 26, 28].

**Table 2:** Draw product line (DPL) variants

Products	$\mathtt {{{Base}}}$	$\mathtt {{{Line}}}$	$\mathtt {{{Rect}}}$	$\mathtt {{{Color}}}$	$\mathtt {{{Wipe}}}$
Product $\mathtt {{P_1}}$	$\checkmark$	$\checkmark$			$\checkmark$
Product $\mathtt {{P_2}}$	$\checkmark$	$\checkmark$		$\checkmark$	
Product $\mathtt {{P_3}}$	$\checkmark$	$\checkmark$	$\checkmark$	$\checkmark$	
Product $\mathtt {{P_4}}$	$\checkmark$	$\checkmark$	$\checkmark$		$\checkmark$
Product $\mathtt {{P_5}}$	$\checkmark$	$\checkmark$			
Product $\mathtt {{P_6}}$	$\checkmark$	$\checkmark$		$\checkmark$	$\checkmark$
Product $\mathtt {{P_7}}$	$\checkmark$	$\checkmark$	$\checkmark$		
Product $\mathtt {{P_{8}}}$	$\checkmark$		$\checkmark$		
Product $\mathtt {{P_{9}}}$	$\checkmark$		$\checkmark$		$\checkmark$
Product $\mathtt {{P_{10}}}$	$\checkmark$		$\checkmark$	$\checkmark$	
Product $\mathtt {{P_{11}}}$	$\checkmark$		$\checkmark$	$\checkmark$	$\checkmark$
Product $\mathtt {{P_{12}}}$	$\checkmark$	$\checkmark$	$\checkmark$	$\checkmark$	$\checkmark$

## Variability extraction data structures and operations

This section introduces basic data structures and operations that will be used in the subsequent sections to explain and formalize trace and dependency extraction.

### Artifacts

We refer to the Java code in Fig. 1 as *Artifacts*. In fact, artifacts can be of many different types, e.g., text strings, *AST (Abstract Syntax Tree)* nodes from a programming language parser, or Ecore objects from for example UML models; for example, in Java such nodes can be classes, fields, methods, statements, etc. Artifacts can contain references to other artifacts, e.g., in Java a statement calling a method references the called method.

### Artifact trees

Artifacts are organized as *Artifact Trees* that represent the hierarchy and the order of the artifacts. In Java, for example, a statement is contained in a method which again is contained in a class.

#### **Definition 6**

(*Artifact Tree*) An *Artifact Tree* is a tree of artifact nodes with arbitrary depth and structure. An *Artifact Node* is a four-tuple $\mathtt {(SN, Artifact, Ordered, Solid)}$. $\mathtt {SN} \in \mathbb {N}$ is the node’s sequence number. $\mathtt {Artifact} \in \mathbb {I}$ is an arbitrary implementation artifact. $\mathtt {Ordered} \in \{true, false\}$ determines whether the children of the node are ordered. $\mathtt {Solid} \in \{true, false\}$ determines if the node is considered to be part of the tree or just a placeholder to keep a path to its children.

A node’s sequence number is initially 0 and for children of unordered nodes it remains 0. For ordered nodes, the order of their children matters, for example a method in Java whose children are statements whose order of course matters. The sequence number is necessary because children of ordered nodes are not uniquely identified by their artifact (e.g., in Java a method can contain the same statement several times at different positions).

A solid node is considered part of the tree, whereas non-solid nodes are just placeholders to keep a path to the root of the tree for solid nodes further down in the tree and to preserve the tree structure for solid nodes. Therefore every leaf node in an artifact tree must always be a solid node. Initially in a product variant every node is solid.

**Fig. 5:**
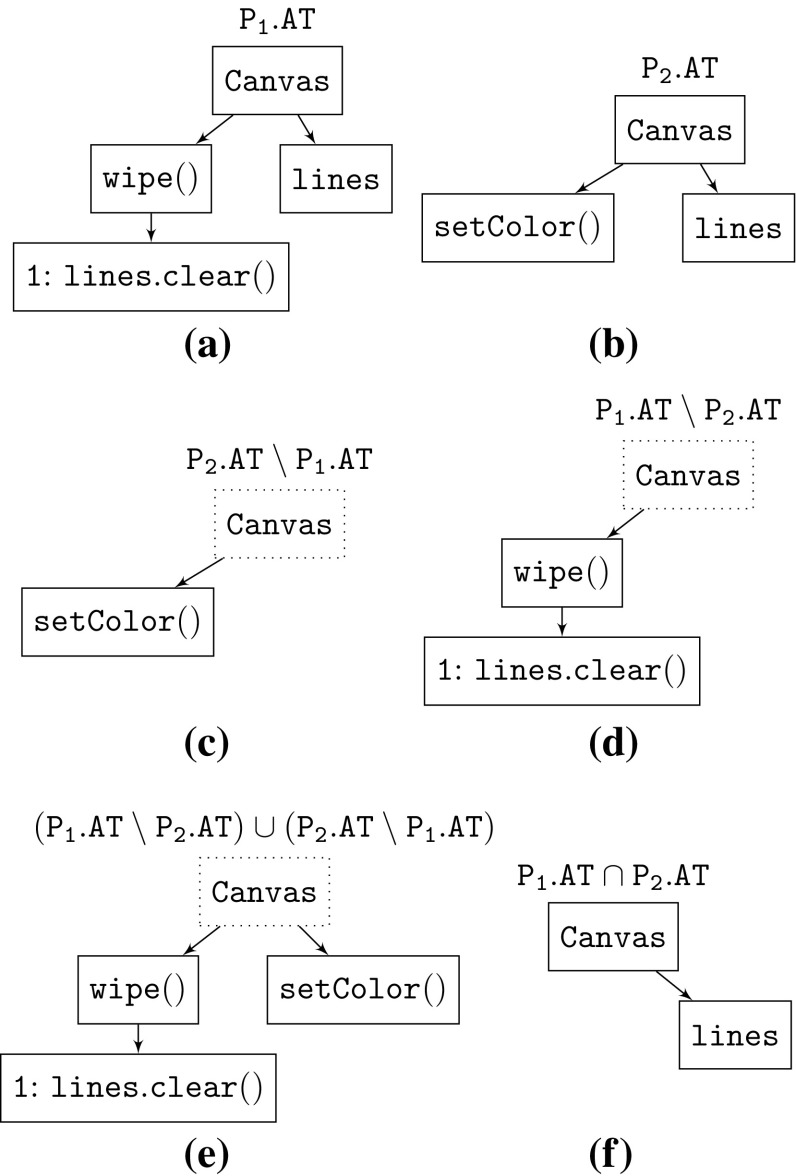
Minus (**c**, **d**), Union (**e**), and Intersection (**f**) operations between artifact trees of product $\mathtt {{P_1}}$ (**a**) and Product $\mathtt {{P_2}}$ (**b**)

For example, Fig. 5a, b show the artifact trees for the class Canvas of product variants $\mathtt {{P_1}}$ and $\mathtt {{P_2}}$, respectively. Node Canvas represents a Java class. It is a solid unordered node which means the order of its children wipe() and line is irrelevant. The node wipe, however, represents a method and therefore is an ordered node, which is why its child lines.clear() has a sequence number of one.

### Operations on artifact trees

To be able to compare and combine artifact trees, we define a number of operations on trees that resemble their set counterparts.

#### **Definition 7**

(*Artifact Node Equivalence*) Two artifact nodes $n_1$ and $n_2$ from two different artifact trees are equivalent ($n_1 \equiv n_2$) iff their sequence numbers are equal, their artifacts are equal, and their parent nodes are, respectively, equivalent.

#### **Definition 8**

(*Artifact Tree Subset Operator*) An artifact tree $AT_1$ is a subset of another artifact tree $AT_2$ ($AT_1 \subseteq AT_2$) iff for every solid node in $AT_1$ there is an equivalent solid node in $AT_2$.

#### **Definition 9**

(*Artifact Tree Intersection Operator*) An artifact tree *AT* is the intersection of two other artifact trees $AT_1$ and $AT_2$ ($AT = AT_1 \cap AT_2$) iff $AT \subseteq AT_1$ and $AT \subseteq AT_2$ and for every solid node in $AT_1$ for which there is an equivalent solid node in $AT_2$ there is also an equivalent solid node in *AT*.

#### **Definition 10**

(*Artifact Tree Difference Operator*) An artifact tree *AT* is the difference of two other artifact trees $AT_1$ and $AT_2$ ($AT = AT_1 \setminus AT_2$) iff for every solid node in $AT_1$ for which there is no equivalent solid node in $AT_2$ there is an equivalent solid node in *AT* and $AT \subseteq AT_1$.

#### **Definition 11**

(*Artifact Tree Union Operator*) An artifact tree *AT* is the union of two other artifact trees $AT_1$ and $AT_2$, denoted with $AT = AT_1 \cup AT_2$, iff $AT_1 \subseteq AT$ and $AT_2 \subseteq AT$ and for every solid node in *AT* there is an equivalent solid node in $AT_1$ or $AT_2$ or in both.

#### **Definition 12**

(*Artifact Tree Cardinality*) The cardinality |*AT*| of an artifact tree *AT* is the number of solid nodes in *AT*.

An example of these operations is given in Fig. 5 by means of product variants $\mathtt {{P_1}}$ (Fig. 5a) and $\mathtt {{P_2}}$ (Fig. 5b), considering only the artifacts of class Canvas for simplicity. Solid nodes are depicted with a solid border and non-solid nodes with a dotted border. Figure 5d shows the result of $\mathtt {{\mathtt {{P_1}}}.AT \setminus {\mathtt {{P_2}}}.AT}$. Those artifacts being unique to $\mathtt {{\mathtt {{P_1}}}.AT}$ remain solid while the common artifacts do not. The intersection operation is shown in Fig. 5f which depicts the result of $\mathtt {{\mathtt {{P_1}}}.AT \cap {\mathtt {{P_2}}}.AT}$ containing all the solid artifacts being common to $\mathtt {{\mathtt {{P_1}}}.AT}$ and $\mathtt {{\mathtt {{P_2}}}.AT}$. Figure 5e shows the union of the two artifact trees in Fig. 5d, c.

Note that, after performing such operations on artifact trees, they will likely not be *well formed* (e.g., in the case of source code *well formed* could mean compilable) anymore on their own. For example the artifact tree in Fig. 5d is missing the declaration of the referenced variable lines and would therefore depend on another artifact tree containing that declaration. This is what will be discussed in Sect. 6 as the extraction of dependencies between modules.

This simplified example shows possible artifact trees for the case of Java source code, which are similar to ASTs, in fact, these trees were derived from ASTs generated by the Java compiler. However, the used artifact trees can also represent, for example, UML diagrams. When using the Eclipse Modeling Framework (EMF) [36], such diagrams are stored in an Ecore data structure which, again, is a tree structure and thus fits perfectly our concept of artifact trees.

### Ordered nodes and sequence graphs

For ordered nodes, a trace is more than just the information of whether an artifact is required for the implementation of a module or not. In addition, the ordering of the artifacts must be considered. For example, the implementation of a certain module could be reflected in the change of the order of artifacts, and when merging the artifacts of an ordered node that stem from different traces it is necessary to know in what order they must be merged. Therefore, for every set of equivalent ordered nodes a sequence graph is maintained.

#### **Definition 13**

(*Sequence Graph*) A *sequence graph* is a directed, acyclic graph with exactly one start node and exactly one end node. Transitions between nodes are labeled with the child artifact nodes of the ordered node the sequence graph belongs to.

A sequence graph holds information equivalent to a partial order relation describing the order of the nodes’ direct children among all traces. Every possible path from the start node to the end node describes a possible ordering and contains every child artifact node exactly once. The nodes of the sequence graph themselves do not contain any information.

Prior to the comparison of artifact trees, we align every new ordered node’s children to the corresponding sequence graph. If no such sequence graph exists, then a new one is created. During the alignment, the sequence numbers of the children of every new ordered node are updated in such a way that the corresponding sequence graph (i.e., the respective partial order relation) is not violated (remember: nodes’ sequence numbers determine, in addition to the nodes’ artifacts, whether two nodes are considered equivalent) and a cost function is minimized. For our purpose, we use a cost function that minimizes the number of new unique sequence numbers assigned, i.e., the cost function maximizes the number of matched nodes, meaning as many nodes as possible with an artifact equal to an already-existing node’s artifact in the sequence graph are assigned the same sequence number if possible without violating the sequence graph (i.e., the underlying partial order relation). After the alignment, the sequence graph is updated to reflect the newly learned (preserving all original) orderings of artifacts.

**Fig. 6:**
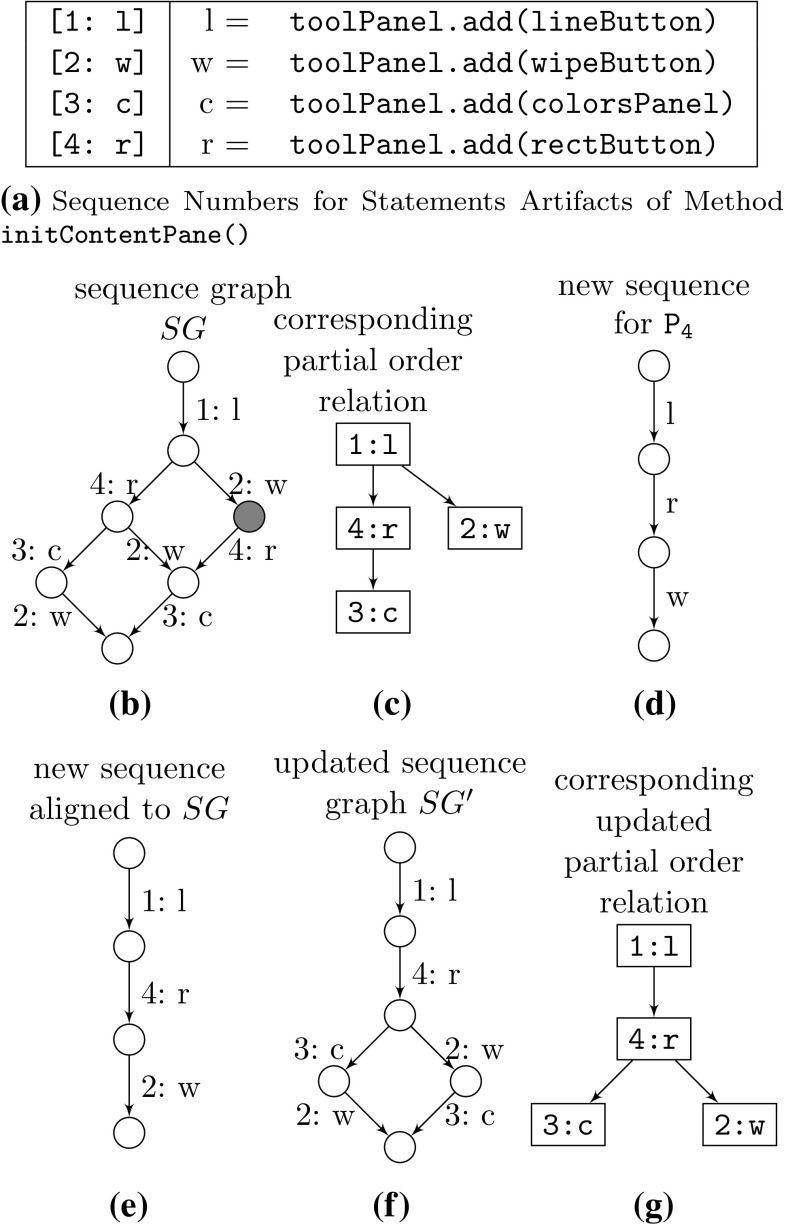
Draw sequence graph example

An example from our set of drawing applications of such a sequence graph, the alignment of a new artifact sequence, and the subsequent update of the sequence graph are shown in Fig. 6. The sequence graph *SG* for the ordered node representing method initContentPane() in class Main expressing the orders of the statements for the initial three product variants is shown in Fig. 6b and the corresponding partial order relation in Fig. 6c. The sequence numbers that were assigned to the statement artifacts are listed in Fig. 6a along with their respective abbreviations so that they fit into the figures. The sequence graph *SG* so far expresses that node [1:l] (i.e., the node with artifact toolPanel.add(lineButton) and sequence number 1) always goes first and that node [3:c] always comes after [4:r], everything else is undetermined.

When adding new product variant $\mathtt {{P_4}}$, its statements of method initContentPane() (shown in Fig. 6d, initially without sequence numbers assigned) must first be aligned to *SG* (i.e., a sequence number is assigned to each statement so that *SG* is not violated and the chosen cost function—here the number of newly introduced sequence numbers—is minimized). The alignment is shown in Fig. 6e. No new sequence numbers needed to be introduced as all artifacts were already known. However, the order between these known artifacts was not fully known. Therefore, after this alignment the sequence graph *SG* is updated to reflect the new knowledge obtained from $\mathtt {{P_4}}$ that the button for rectangles [4:r] must be added before the button for wiping the canvas [2:w]. Therefore, the rightmost node of *SG* (marked in black in Fig. 6b) can simply be removed, resulting in the updated sequence graph $SG'$ shown in Fig. 6f, again with the corresponding partial order relation in Fig. 6g. The order between the colors panel and the wipe button still cannot be determined.

This alignment process is repeated for every pair of ordered nodes whenever two artifact trees are compared. Note that the sequence graph shrinks in size the more order information becomes available, as nodes are removed from the sequence graph whenever the order between artifacts becomes more determined. When the order between artifacts is fully determined only one path through the sequence graph remains (i.e., the sequence graph is simply a list) describing exactly the one valid order.

## Trace extraction

The trace extraction is based on five rules. Given two product variants $\mathtt {{A}}$ and $\mathtt {{B}}$: The first two rules quickly isolate modules to which certain implementation artifacts *at least* trace (to compute *Minimal* traces).

1. Common artifacts *at least* trace to common modules.
2. Artifacts in A and not B *at least* trace to modules that are in A and not B, and vice versa.

However, in rare cases two product variants can have code in common without having modules in common. This is the case for non-unique or disjunctive traces, where implementation artifacts trace to different disjunctive modules, i.e., the artifacts are included in a variant if at least one of the modules is included. The next three rules help to deal with such cases by identifying to which modules artifacts certainly *cannot* trace (*Not* traces) and to which they can *at most* trace (*Maximal* traces).

3. Artifacts in A and not B *cannot* trace to modules that are in B and not A, and vice versa.
4. Artifacts in A and not B can *at most* trace to modules that are in A, and vice versa.
5. Artifacts in A and B can *at most* trace to modules that are in A or B.

**Fig. 7:**
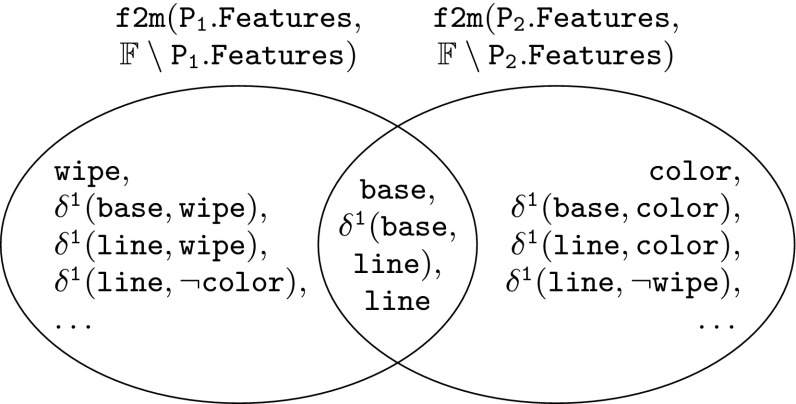
Modules comparison of products $\mathtt {{P_1}}$ and $\mathtt {{P_2}}$

These rules require the comparison of module sets as well as different artifact trees. The comparison of the module sets is based on simple set operations. Figure 7 shows the comparison of the modules of product variants $\mathtt {{P_1}}$ and $\mathtt {{P_2}}$. The comparison of artifact trees is performed in a similar fashion using the operators defined in the previous section. The extracted information is stored in the form of associations between modules and artifacts that trace to these modules.

### **Definition 14**

(*Association*) An *Association* $\mathtt {A} \in \mathbb {A}$ is a relation $\mathtt {(M, AT)}$ of a four-tuple of module sets $\mathtt {M = (Min,}$ $\mathtt {All, Max, Not)}$ and an artifact tree $\mathtt {AT}$.

Associations are used by the extraction algorithm as containers that are incrementally filled. The module sets *A*.*M* are essentially lower and upper bounds on the modules that are realized (or not realized) by the artifact tree *A*.*AT* of association *A*. *A*.*M*.*Min* is the set of modules to which the association’s artifacts *at least* trace (see Rules 1 and 2 above), *A*.*M*.*Not* is the set of modules to which the artifacts *cannot* trace (Rule 3), *A*.*M*.*Max* is the set of modules to which the artifacts can *at most* trace (Rules 4 and 5) and *A*.*M*.*All* is the set of all modules with which the artifacts have ever been associated (needed for computing *A*.*M*.*Max*).

The *Trace Extraction* incrementally refines an initially empty set of associations according to new information that becomes available when adding a new input product variant, i.e., given a product variant $P \in \mathbb {P}$ and a set of associations $A \in \mathbb {A}$, it produces a refined set of associations $A'$. Henceforth, we refer to such a set of extracted associations as *database*.

### **Definition 15**

(*Trace Extraction*) $Extraction: \mathbb {P} \times 2^\mathbb {A} \mapsto 2^\mathbb {A}$ where $\mathbb {P}$ denotes the universe of all product variants and $\mathbb {A}$ denotes the universe of all associations.

The high-level pseudo code for the extraction process is shown in Algorithm 1. Lines 3 to 7 do the initialization. Lines 9 to 27 iterate over every association *a* in *A*, update its modules with new features (Line 11) and perform the alignment and sequencing of matching ordered nodes (Line 13). Then the associations are updated according to the Rules 1 to 5 stated above (Lines 15 to 23). Association *a* in *A* is compared to the new association $a_{new}$ by computing an association for the intersection $a_{int}$ and updating the old association *a* and the new association $a_{new}$ accordingly. Line 26 adds the intersection association $a_{int}$ to the set of associations $A_{new}$ to be returned. Lastly, the remainder of the new association $a_{new}$ is added to the set in Line 30 and then returned.
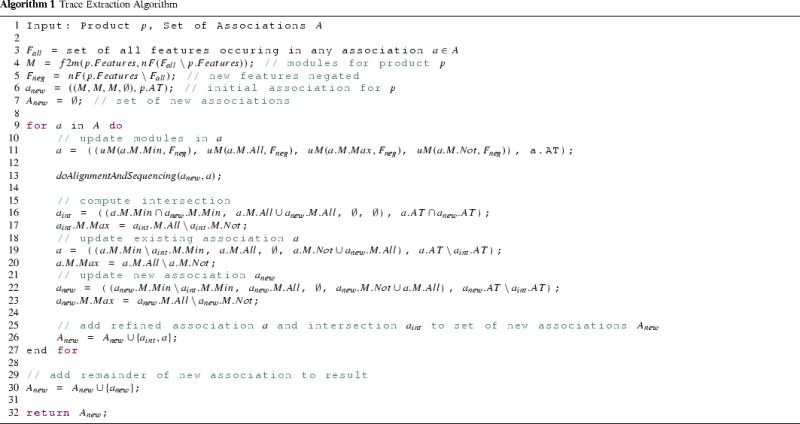


Note that our trace extraction approach captures exactly the variability present in the used input product variants. This is ideal for sets of well-maintained product variants. However, if the variants have been maintained inconsistently and therefore have diverged from each other (e.g., bug fixes applied to only some of the variants or features implemented slightly differently) also all the inconsistencies are captured. This does not mean that the extracted traces are wrong, but it simply means that they might be more complex and difficult to interpret as for example different traces (i.e., associations) for different implementations of the same feature are extracted.

## Dependency extraction

This section describes the dependency graph extraction from a set of associations $\mathbb {A}$. A dependency graph represents a set of constraints on possible feature combinations imposed by their implementation. They can be regarded as simple variability models of a system describing what combinations of features can be selected to form product variants.

### **Definition 16**

(*Dependency Graph*) A dependency graph $DG: \mathbb {A} \times \mathbb {A} \mapsto \mathbb {N}$ is a function that maps to every ordered pair of associations $(A_1, A_2)$ a number denoting how strongly $A_1$ depends on $A_2$.

$$\begin{aligned} DG(A_1 \in \mathbb {A}, A_2 \in \mathbb {A}) \\ =\,|\{ N_1 \mid \exists {} N_2 \in A_2.AT&: N_1 \in A_1.AT \wedge {} \\&N_1.\textit{Solid} = N_2.\textit{Solid} = \textit{true} \wedge {} \\&\textit{child}(N_1.\textit{Artifact}, N_2.\textit{Artifact}) \}|\\ +\\ |\{ N_1 \mid \exists {} N_2 \in A_2.AT&: N_1 \in A_1.AT \wedge {} \\&N_1.\textit{Solid} = N_2.\textit{Solid} = \textit{true} \wedge {} \\&\textit{uses}(N_1.\textit{Artifact}, N_2.\textit{Artifact}) \}| \end{aligned}$$

The dependency graph is computed based on dependencies between implementation artifacts in the artifact trees of associations. The first summand expresses the dependencies of child artifacts in $A_1$ on their parent artifacts in $A_2$ (e.g., a method in $A_1$ depends on its containing class in $A_2$), the second summand expresses the dependencies of artifacts in $A_1$ on other artifacts in $A_2$ they use in some way (e.g., a statement in $A_1$ calling a method in $A_2$ depends on that method). From the dependencies between artifact trees, we derive dependencies between associations and can thus further derive dependencies between modules. In other words, based on artifact dependencies which are given (e.g., by source code constraints), we compute dependencies between associations and therefore between the modules contained in the associations.

Just like feature models, dependency graphs can be represented as a set of constraints in propositional logic. Every dependency, i.e., every edge in the graph, represents one constraint. The propositional logic representation of a dependency $DG(A_1, A_2)$ between two associations $A_1$ and $A_2$ is

$$\begin{aligned} \qquad \qquad \bigvee \limits _{from \in A1.Min}from \Rightarrow \bigwedge \limits _{to \in A2.Min}to \end{aligned}$$

Note that if for any association *A*, there are no minimal modules, i.e., $A.\textit{Min} = \emptyset$, then we use $A.\textit{Max}$ instead. The propositional logic expression for the whole dependency graph *DG* is simply the conjunction of all its individual dependencies.

**Fig. 8:**
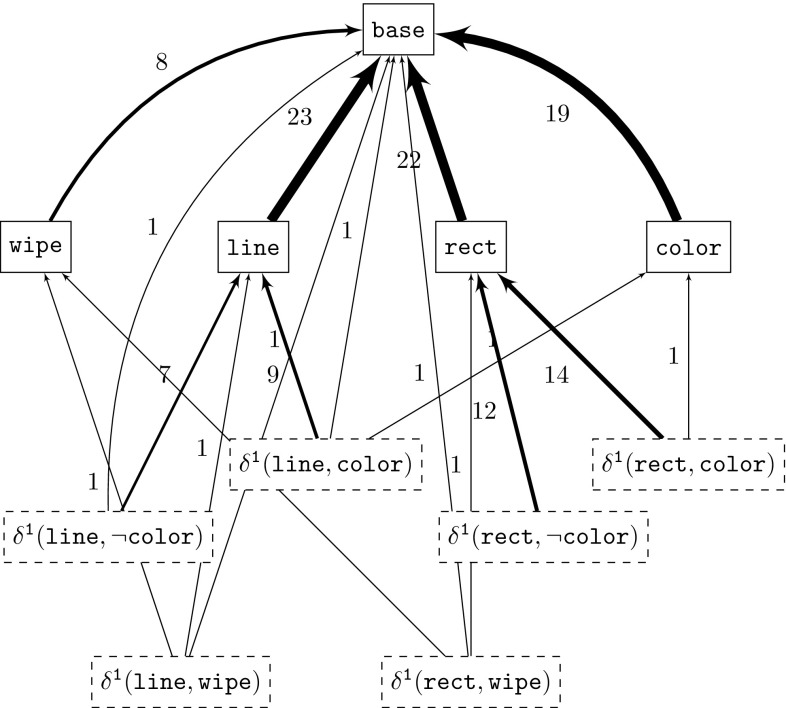
Dependency graph for draw case study

Figure 8 shows the dependency graph for our drawing application running example after having used all its twelve product variants as input to the extraction process. The nodes in the dependency graph are labeled with the corresponding association’s lowest order modules for sake of brevity. Only the *Min* modules are considered here, unless there are no *Min* modules in an association in which case the *Max* modules are used. Higher-order modules are not depicted for space reasons and to avoid clutter. Base modules are depicted as solid boxes while derivative modules are depicted as dashed boxes. Arrows between nodes represent a dependency. The number on an arrow as well as its thickness denote the strength of a dependency, that is, for associations $A_1$ and $A_2$ this number corresponds to $DG(A_1, A_2)$. For better readability the self-dependencies are not shown. We found that for base modules, they were always by far the strongest, for example the association with module $\mathtt {{{base}}}$ depends on itself with a strength value of 166, or $\mathtt {{{line}}}$ depends on itself with a strength of 93. For associations with derivative modules, the self-dependencies were sometimes matched or even surpassed by the dependencies to the corresponding base modules, e.g., $\mathtt {\delta {}^{1}({rect,\lnot {}color}})$ depends on itself with a strength of 14 and on $\mathtt {{{rect}}}$ with a strength of also 14. This could be a good indication for the extracted traces being correct as this is evidence of high cohesion within traces.

Based on the dependency graph, we can make the following observations:

- Aside from the self-dependencies, the strongest dependencies are toward the association containing the base module $\mathtt {{{Base}}}$ which corresponds to feature $\mathtt {{{Base}}}$ which in turn is the root node of the DPL feature model.
- The most and the strongest dependencies originate from the associations containing the other base modules (e.g., $\mathtt {{{Wipe}}}$, $\mathtt {{{Line}}}$, $\mathtt {{{Rect}}}$, or $\mathtt {{{Color}}}$).
- Associations with derivative modules depend mostly on the associations that contain the corresponding base modules, e.g., the derivative module $\mathtt {\delta {}^{1}({rect,color}})$ depends on the two associations containing module $\mathtt {{{rect}}}$ and module $\mathtt {{{color}}}$.
- Associations with negative features never have dependencies to associations with positive versions of the same features; otherwise, this association could never be included in a product variant because the dependency would always be violated.

We argue that these observations, in general, are good indications for the correctness of the extracted traces. What is also of interest is that the extracted dependency graph is consistent with the feature model, i.e., the constraints imposed by the feature model imply the constraints of the dependency graph. Recall that a feature model denotes the set of valid product variants. A good indication that the extracted information is correct is when none of the variants from a feature model violate any of the dependencies of the dependency graph. The feature model, however, might impose additional constraints to restrict the set of possible product variants further, i.e., the constraints of a feature model must imply the constraints of the dependency graph. For example, the feature model requires at least one of the two features $\mathtt {{{LINE}}}$ and $\mathtt {{{RECT}}}$ to be present in every product variant because otherwise the variant would make no sense. However, this is not a requirement in the dependency graph.

Nonetheless, in cases where there is no feature model available, as for example when reverse engineering a set of product variants into a product line, the dependency graph can provide a good starting point for reverse engineering a feature model. When only considering the associations in the dependency graph that correspond to base modules, the graph already resembles very much the DPL feature model in Fig. 4. We argue that the additional constraints that such dependency graph provides can be useful for feature model reverse engineering approaches as our recent work suggests [5].

## Evaluation

This section evaluates the proposed approach using *six different case studies*.

### Methodology

As a first proof of correctness, we check that the extracted *dependency graphs are consistent with the feature models*. In a next step, the *consistency of the extracted traces with the given product variants* is evaluated by using them to reconstruct product variants and comparing them to the originals. Finally, *detailed metrics for the extraction process* are explained and shown for each of the case studies. The section concludes with an analysis of the results.

We follow the following overall methodology for the evaluation:

- We briefly introduce the used case studies. Each case study consists of a set of product variants. Additionally, every case study (except for one) also comes with a feature model that expresses its product variants.
- Using the case studies that came with a feature model, we evaluate the correctness of the extracted dependency graphs. The detailed methodology is further explained in Sect. 7.3.
- For every case study, we verify the correctness of the extracted traces by using them to reconstruct product variants and comparing them to their original counterparts. Again, the detailed methodology is further explained in Sect. 7.4.
- Finally, we show some detailed metrics about the extraction process and discuss them.

### Case studies

An overview of the used case studies is shown in Table 3. The following subsections explain them in detail. All of these case studies are implemented in Java. Hence, artifacts include for example classes, methods, fields, and statements. The artifact trees for the case studies were obtained using a Java compiler’s ability to parse Java source code and generate ASTs from it that can be retrieved through the provided APIs. Java compilers that provide such functionality are for example the Java Compiler Tree API^1^ that comes with the Oracle JDK or the Eclipse Java Development Tools^2^.

**Table 3:** Case studies overview

Case study	#F	#P	LoC	#Art
DPL	5	12	287–473	487
VOD	11	32	4.7–5.2K	5.5K+
ArgoUML	11	256	264–344K	200K+
ZipMe	7	32	5–6.2K	6.2K+
GOL	15	65	874–1.9K	1.3K+
MA	13	5	35–59K	88K+

#F: Number of Features, #P: Number of Products, LoC: Range of Lines of Code, #Art: Number of Distinct Artifacts

Our case studies were selected to range from *ideal* scenarios where product variants are well maintained to *worst* scenarios where variants have significantly diverged. The first five cases represent a more idealistic scenario because they come from SPL examples and are thus better maintained variants. However, please note that many of these case studies were not designed as product lines from the very beginning but refactored at a later time, which is why we believe that they are representative to a certain degree even beyond product lines. The last case study ModelAnalyzer (MA) represents the other extreme. MA has evolved over a period of more than five years. It was developed by many students and engineers for different purposes and goals and with different coding styles. In addition, only five variants are available which could make it harder for the extraction to achieve good results since the extraction process can only perform few iterations to refine the traces.

#### Draw product line (DPL)

The *DPL* case study is a set of simple drawing applications that was refactored into an SPL and used as a running example throughout this paper.

#### Video on demand (VOD)

**Fig. 9:**
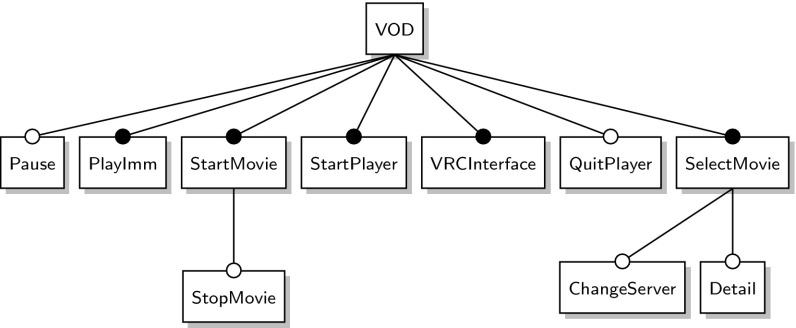
VOD feature model

*Video on demand (VOD)* is an SPL for video-on-demand streaming applications. It started out as a single product which was then refactored into a product line. It supports eleven features of which six appear in every variant. The feature model is shown in Fig. 9. It allows for the generation of 32 product variants.

#### ArgoUML

**Fig. 10:**
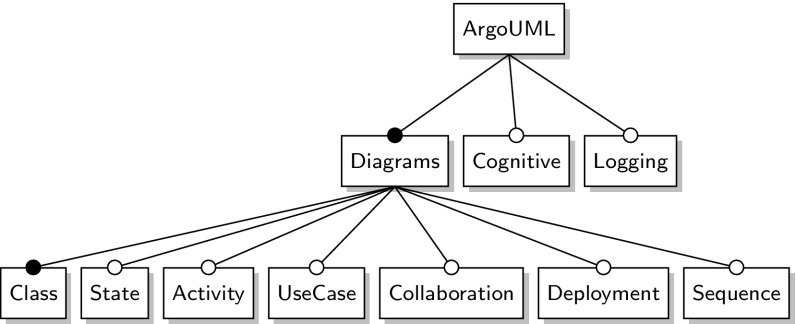
ArgoUML feature model

The largest case study *ArgoUML* is an open-source UML modeling tool that was refactored into an SPL [4, 11]. It has eleven features of which three appear in every product variant. According to its feature model, shown in Fig. 10, there are 256 product variants.

#### ZipMe

**Fig. 11:**
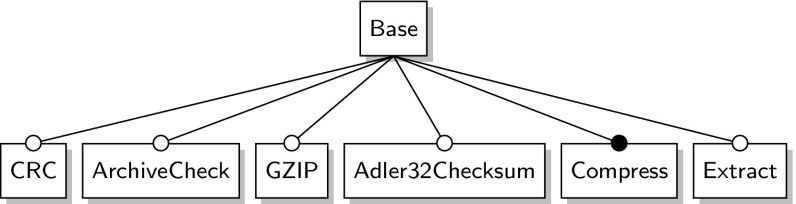
ZipMe feature model

*ZipMe* is a compression software with seven features and 32 different product variants. Its feature model is shown in Fig. 11.

**Fig. 12:**
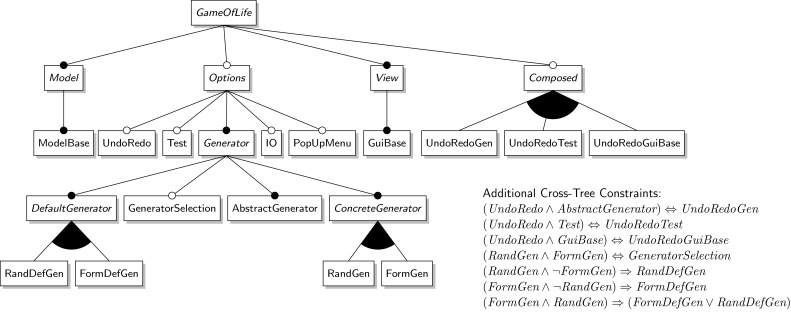
GOL feature model

#### Game of life (GOL)

*Game Of Life (GOL)* is a product line for the game of the same name. It has 23 features in total of which 8 are declared as *abstract features* (shown in italics in the feature model in Fig. 12) which are used to help structure the feature model but that do not have artifacts associated with them. So effectively there are 15 different features in this case study.

#### ModelAnalyzer (MA)

The last case study *ModelAnalyzer (MA)* is a consistency checking and repair technology [14]. It is not an SPL, but rather its variants were created through copying from existing variants and then developed independently of each other by different engineers who each had their own goals. In total, we had five different variants available with 13 features altogether. Since MA is not an SPL there is no feature model available. It is unknown how many possible variants there would be and what features are mandatory or optional. Nonetheless, MA can be used in our extraction process as pointed out in the introduction. Consequently, however, in this case, the extracted dependency graph cannot be compared to a feature model. This is how we expect scenarios to be in practice and where the extracted dependency graph is most useful as it can be used to create a feature model.

The information about the variants (i.e., their source code and the features they implement) were obtained through interviews with the respective developers. Difficulties were for example that some developers had partially implemented features from other variants they copied from and just left them in their unfinished state because they did not use these features anyway. Also common names for features had to be established because different developers used different names for the same features. This is only to emphasize how difficult a case study ModelAnalyzer represents for the extraction process. Note that the product variants were used exactly as provided without any preprocessing, i.e., *no* prior cleanup of the variants was performed at all.

### Dependency graphs validation

**Fig. 13:**
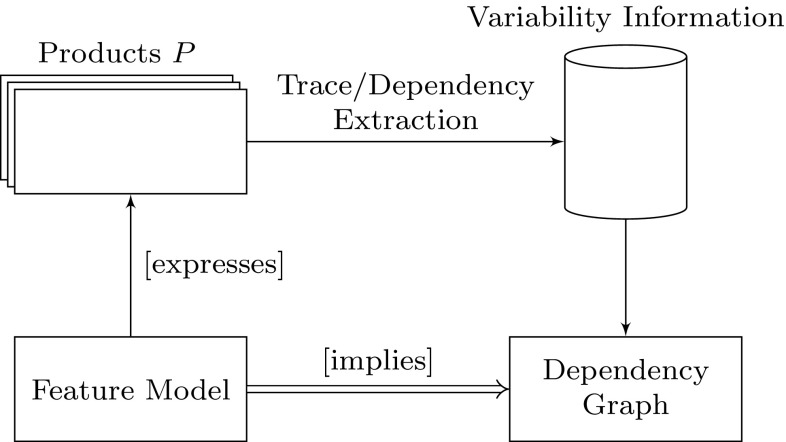
Dependency graphs validation

This section compares the extracted dependency graphs to the corresponding feature models as was done for the DPL running example in Sect. 6, except for the MA case study for which no feature model is available. The comparison between extracted dependency graphs and feature models allows us to verify that the extracted traces and their dependencies (which represent the implementation variability of the analyzed systems) adhere to the respective feature model (representing the design variability) which would be a strong indication that the extracted traces are correct. The comparison is based on the fact that a dependency graph *DG* as well as a feature model *FM* are both comprised of sets of constraints that can be represented as propositional logic formulas. A dependency graph represents static dependencies between artifacts and therefore must hold for every valid (i.e., well-formed) product variant. For a feature model to be variability safe [5], it must guarantee that all product variants it describes are well formed (but it does not need to denote all possible well-formed product variants). The feature model must therefore imply the dependency graph (i.e., $FM \implies DG$). The whole process is shown in Fig. 13.

**Fig. 14:**
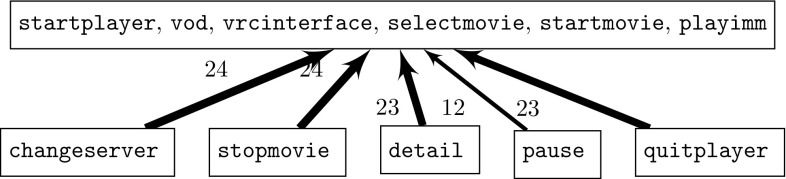
Dependency graph for VOD case study

For space reasons only the dependency graphs for the *DPL* and *VOD* case studies are shown as these graphs can become quite large. In Fig. 14, the dependency graph for the VOD case study is shown. It is much simpler than the dependency graph of the DPL case study system. Since for VOD six features are mandatory and always present in every variant they all appear in one association which is also the one with the strongest dependencies. All the other associations depend on it. In terms of modules and the corresponding features, this again is reflected in the feature model in Fig. 9.

The dependency graphs for the ArgoUML and GOL case studies were also consistent with the respective feature models. For the ZipMe case study, however, there was a violation. The ZipMe feature model denotes product variants with feature $\mathtt {{{GZIP}}}$ but without feature $\mathtt {{{CRC}}}$. In the dependency graph however, the feature $\mathtt {{{GZIP}}}$ requires feature $\mathtt {{{CRC}}}$. Upon closer investigation, we found that this is because $\mathtt {{{GZIP}}}$ requires a type defined in $\mathtt {{{CRC}}}$. This means the feature model of the ZipMe case study, as provided by its developers, allows for the generation of erroneous product variants. This is an instance of the optional feature problem [21].

### Trace correctness validation

**Fig. 15:**
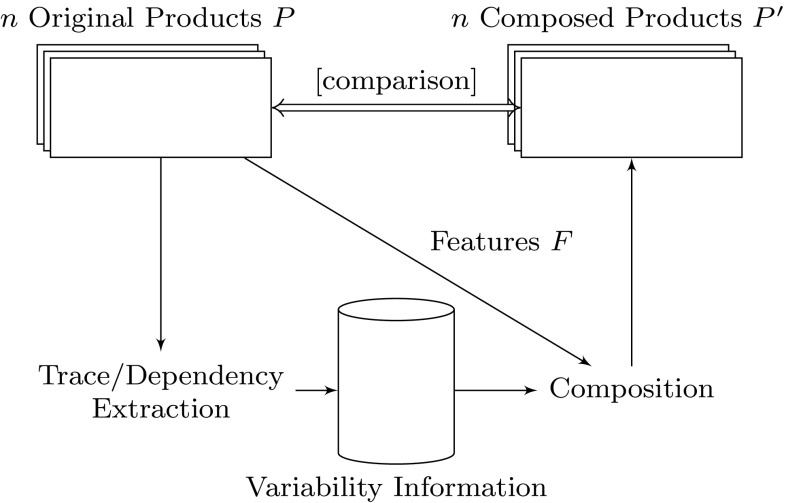
Trace extraction validation

We also evaluated the correctness of the extracted trace information by using it to generate product variants and comparing these variants to the original counterparts. This is to show that the extracted traces are correctly representing the variability within the product variants. For the generation of product variants from the extracted variability information, we used the composition approach of our previous work [15]. For this purpose for every case study, all the *n* available product variants were used as input to the extraction. Then, the extracted traces were used to recompose the product variants. An overview of the whole process is shown in Fig. 15. For every product variant, the number of missing and surplus implementation artifacts were counted and averaged over the number *n* of variants. Let *P* be the original product variant and $P'$ the corresponding (i.e., with the same set of features) composed product variant.

#### **Definition 17**

$$\textit{Surplus} (\%) = \frac{|{\mathtt {{P'}}}.AT \setminus {} {\mathtt {{P}}}.AT|}{|{\mathtt {{P}}}.AT|}$$

#### **Definition 18**

$$\textit{Missing} (\%) = \frac{|{\mathtt {{P}}}.AT \setminus {} {\mathtt {{P'}}}.AT|}{|({\mathtt {{P}}}.AT)|}$$

For every case study, these two metrics were at **0** % of the number of implementation artifacts, meaning that the extracted traces were always consistent with the product variants, which is another strong indication that the extracted traces are correct.

### Extraction metrics

The purpose of these metrics is to provide more insight into the extraction process and the quality of the extracted variability information.

#### Runtime performance

**Fig. 16:**
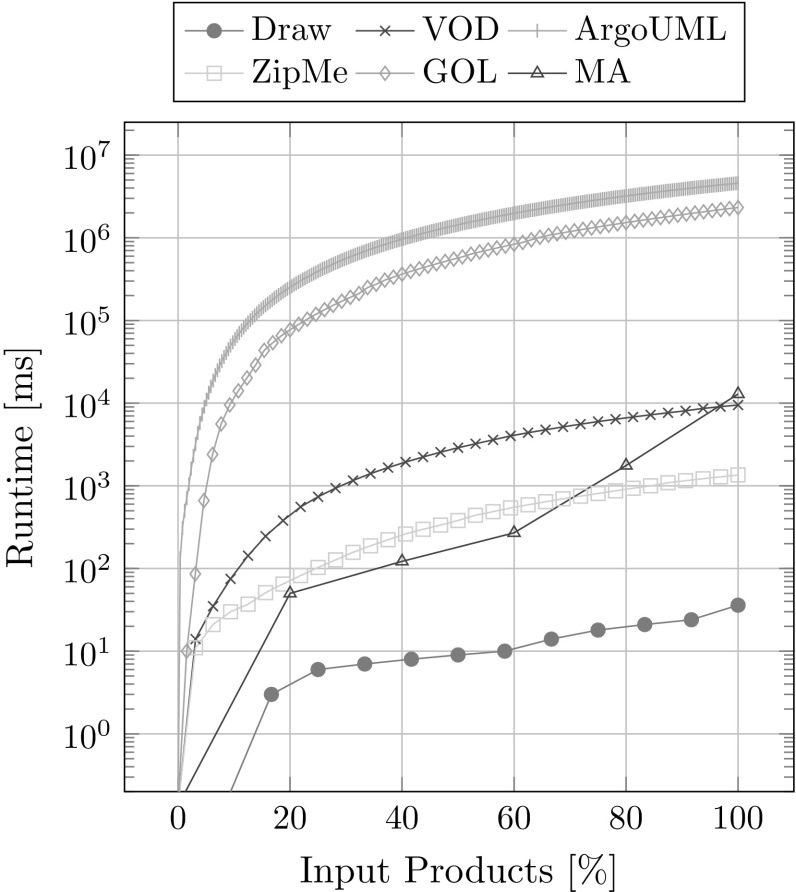
Runtime overview

We measured the runtime of different portions of the extraction process, not including the parsing of the input product variants, on an Intel(R) Core(TM) i7-3770 CPU with 3.4 GHz and 16 GB of main memory. Eight runs were performed each with a randomized order of the input product variants and the results averaged. Figure 16 shows the average runtime after each newly added product variant on a log-axis. Most of the runtime is spent on processing the modules and is therefore dependent on the number of features. This includes computing modules for new input product variants or comparing module sets. The processing of the artifact trees only takes up a relatively small portion of the total runtime as shown in Table 4. This is especially the case for case studies with many features (see GOL), as more features lead to more modules. Lastly, of course a high number of product variants (see ArgoUML) also increases the runtime.

**Table 4:** Average runtime distribution for modules and artifacts processing

Case study	Modules processing (%)	Artifacts processing (%)
DPL	68.7	31.3
VOD	97.9	2.1
ArgoUML	99.1	0.9
ZipMe	88.8	11.2
GOL	99.9	0.0001
MA	98.9	1.1

#### Modules per order

Recall that the order of a module is a measure for the number of interacting features. A module of order *o* represents $o+1$ interacting features. For example, module $\mathtt {\delta {}^{3}({base, line, wipe, color}})$ is of third order which means it represents the interaction of four features. For every order, we compute the total number of modules with that order that are associated with at least one artifact in the final database (i.e., with all available product variants added). This metric is interesting because it tells us up to what order modules actually require artifacts to implement them.

**Fig. 17:**
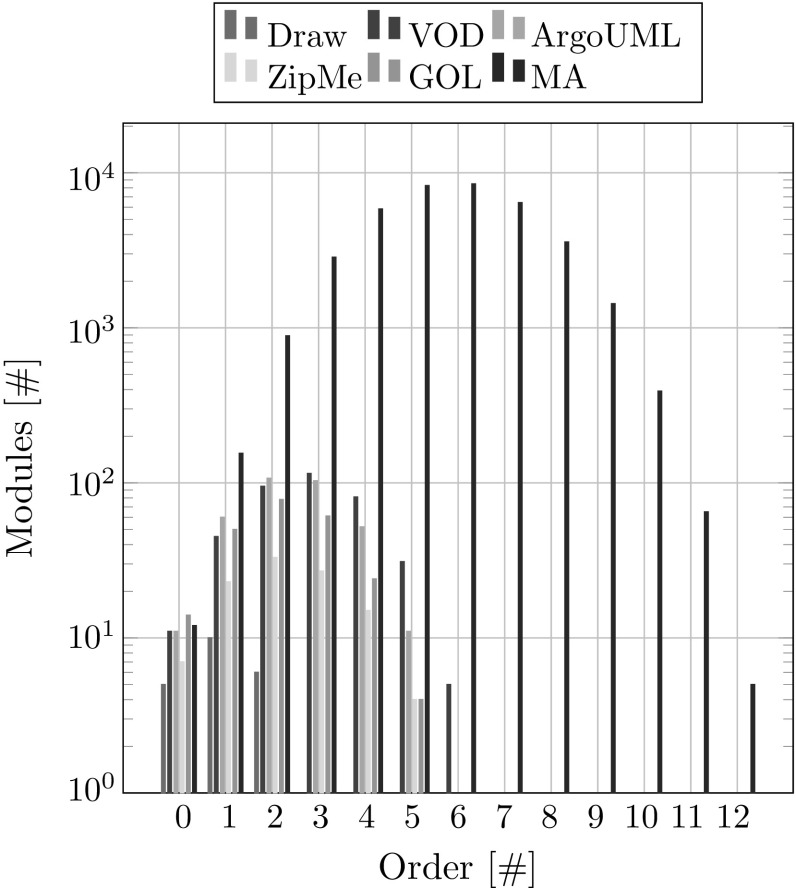
Modules per order overview

The result is shown in Fig. 17. Only the *Min* modules, i.e., *A*.*M*.*Min* for every association *A*, are considered here. This is because the set of modules that the corresponding artifacts can *at most* trace to, i.e., *A*.*M*.*Max*, can become quite large and this metric would lose meaning. Also, in practice, whenever there are *Min* traces available they are the preferable ones, because they are the ones the artifacts most probably trace to. Only when such traces are not available other trace information like where artifacts *cannot* trace (*A*.*M*.*Not*) or where they can *at most* trace (*A*.*M*.*Max*) become really useful.

Given a number of features *n* in a domain, the highest order derivative that can appear in that domain is $n-1$. However, except for the ModelAnalyzer case study none of the highest order derivative modules actually were associated with any artifacts. This is because the number of available input product variants for MA is very small and therefore the extraction could not rule out the possibility of some of the higher-order derivatives containing code. Considering that it is increasingly unlikely for higher-order derivatives to be associated with artifacts, a *threshold* for the maximum order of derivatives could be used. This would reduce the number of modules and hence also reduce the runtime, which, as was shown in the previous subsection, is mostly spent on processing modules.

This can also have an impact on the testing process of sets of product variants. As most higher-order modules do not contain implementation, they need not be covered by tests, which means fewer product variants need to be tested to cover all the modules that actually contain code.

#### Number of artifacts

**Fig. 18:**
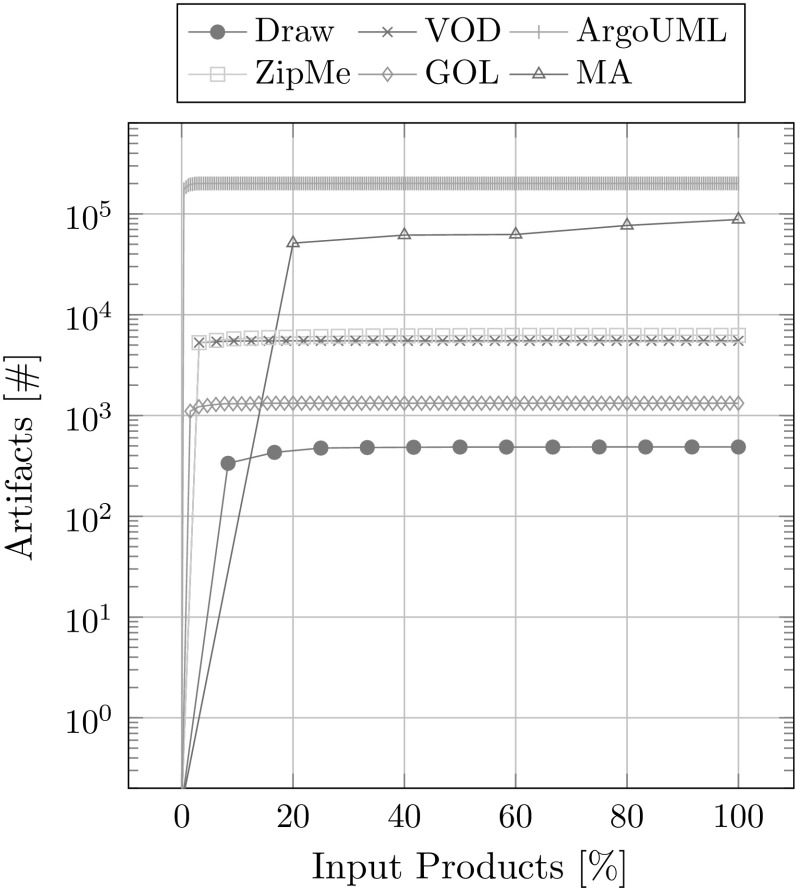
Artifacts overview

The number of artifacts in the database is a simple metric that hints at the size of the database. Again eight runs were performed with a randomized order of input product variants. Figure 18 shows the average number of artifacts after each newly added product. In every case study, it takes only very few input product variants (less than 5) to have almost all the artifacts available. Adding further product variants improves other metrics like the number of associations or the distinguishability (see the following metrics) but do not improve much on the number of available artifacts.

Again this can have implications for testing. In order to achieve high code coverage of test suits only few product variants need to be tested.

#### Number of associations

**Fig. 19:**
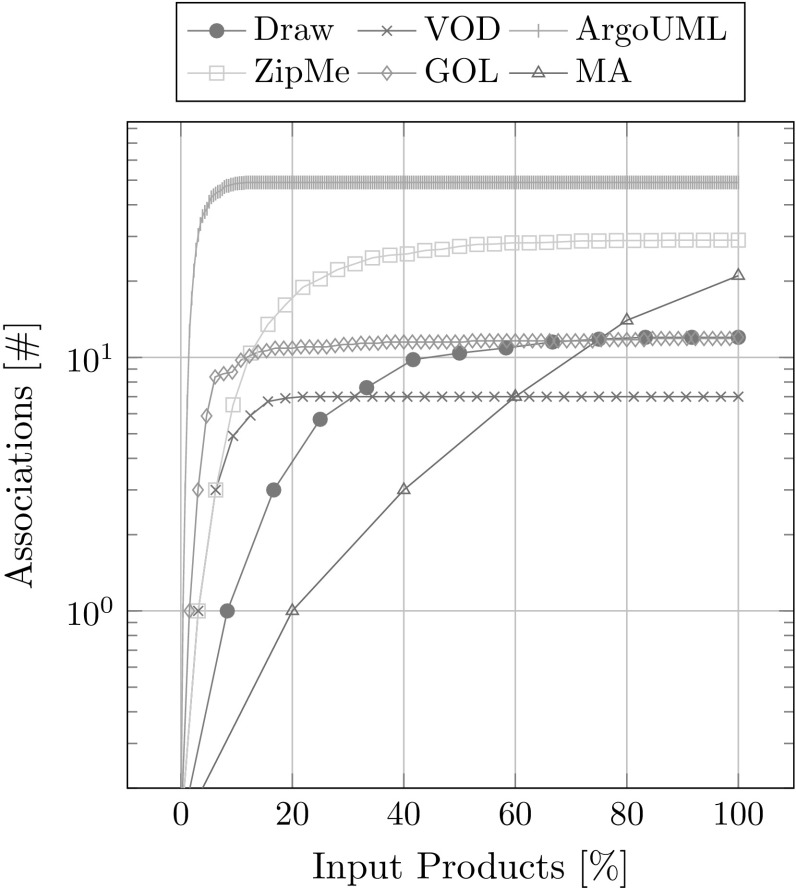
Associations overview

The average number of associations after each newly added product variant over eight runs with randomized order of input product variants is shown in Fig. 19. Similarly as for the number of artifacts also the number of associations increases very quickly already with the first few input product variants, although not with quite as few. However, it keeps increasing steadily with more additional product variants before it finally reaches its peak.

#### Distinguishability

Distinguishability describes the number of modules per association.

##### **Definition 19**

Distinguishability is the average cardinality of all module sets whose respective associations contain at least one artifact and at least one module.

$$\begin{aligned} \textit{Distinguishability} = \frac{1}{n} * \sum _{i=1}^{n}|\textit{association}_i.M| \end{aligned}$$

where *n* is the number of associations that contain at least one artifact and at least one module and $association_i$ is such an association.

**Fig. 20:**
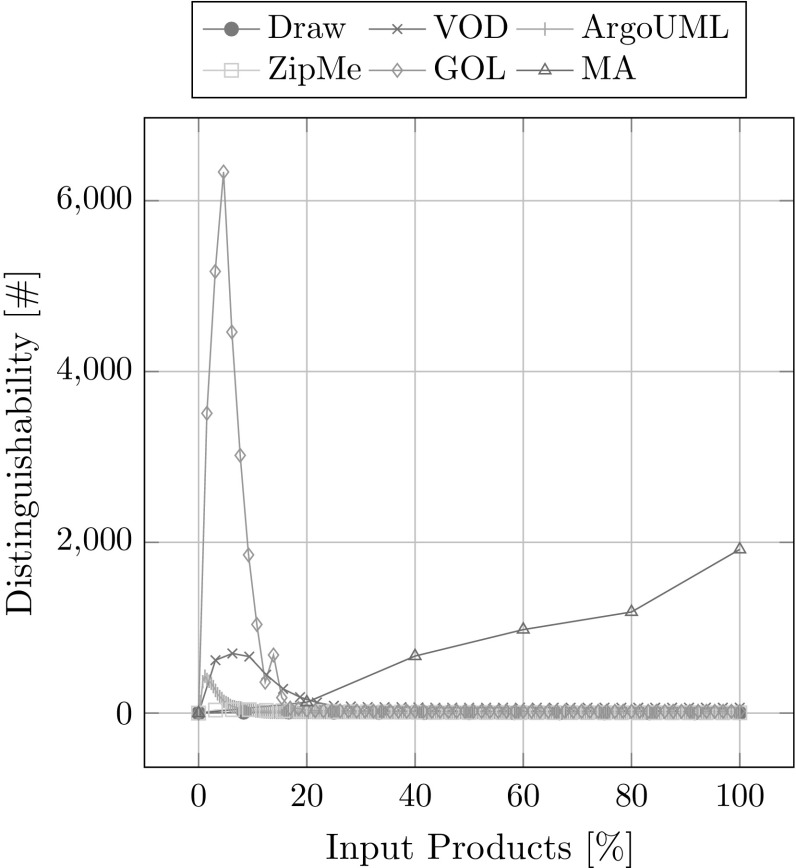
Distinguishability overview

The purpose of this metric is to measure how many modules on average could not be separated because they never appeared without each other in any of the input product variants. The optimal value for this metric would be 1, meaning every association containing at least one artifact would have exactly one module. However, this can only very rarely be achieved due to mandatory features that are present in every product variant or features that can never appear without each other, also known as *atomic sets* [8]. In Table 5, the lower bound for the theoretically best achievable distinguishability (the number of inseparable modules formed by the *n* mandatory features: $=2^n-1$) and the actually achieved distinguishability is shown for every case study with two exceptions: GOL has a more complex feature model so the optimal value is non-trivial to compute, and ModelAnalyzer for which we do not have a feature model available. The *distinguishability* improves with the number of input product variants and is generally worse the more features there are. Again this metric was computed for eight runs with a random order of input product variants and then averaged. As is shown in Fig. 20, the distinguishability first gets worse quickly but then improves steadily with every additional input product variant. This is in contrast with our other metrics. Only the ModelAnalyzer case study does not reach the point where the distinguishability improves because of the small number of available product variants. This means that the average number of modules per association increases with the first few input product variants added. After that critical number of product variants, however, modules become increasingly separated with every additional input product, and it becomes possible to determine more accurately which modules really trace to certain implementation artifacts by ruling out other modules that do not.

**Table 5:** Distinguishability overview

Case study	Lower bound	Achieved
Draw	$2^1 - 1 = 1$	1.9
VOD	$2^6 - 1 = 63$	63.8
ArgoUML	$2^3 - 1 = 7$	7.2
ZipMe	$2^2 - 1 = 3$	3.9
GOL	NA	21.0
MA	NA	1917.2

### Discussion and summary

In summary our metrics show solid results. The dependency graphs and the corresponding feature models do not contradict each other. The extracted traces are consistent with the variability inherent to the used input products. The runtime for adding new products increases with the size of the database. Most of the runtime is taken up by processing modules. It makes sense to introduce a threshold for the maximum order of derivatives that will be computed to decrease the runtime, because most higher-order derivative modules do not have any implementation artifacts. This would also have a positive effect on distinguishability, as there will be fewer modules per association. The number of artifacts in the database as well as the number of extracted associations reach a peak already with very few input products, which means that the presented approach can already function well with just few available input products. Only for achieving a near optimal distinguishability, it is necessary to have a large set of available products.

### Threats to validity

The first threat is in the selection of case studies to represent the problem domain. Our current selection only consists of systems that are implemented in Java. As we use a generic data structure very similar to ASTs to represent the implementation artifacts, and most programming language parsers provide exactly such an AST, we do not expect the results to differ much for other programming languages. Additionally, we were successful in extending our approach to support UML diagrams (in EMF Ecore format) and are currently investigating its application to other types of artifacts like Excel sheets or CAD (Computer-Aided Design) drawings. Unfortunately, however, we could not get access to any realistic and publicly available case studies of any such kind that could be used in our evaluation, but we are constantly looking for them.

Another threat is the selection of our extraction metrics we devised in our evaluation. Most of them are a direct result of our algorithm and can be measured directly (e.g., by simple counting), like the number of associations or the modules per order. These metrics help us to discuss interesting patterns the results, and their implications for our future research and the research domain in general, but they are not critical for the validity of the evaluation of our approach. We simply report on these metrics to give further insights into our approach and a better understanding of the results.

## Related work

Our previous work presented a framework and workflow that combines extraction and composition support for clone-and-own [15]. Also, we have applied our extraction work to a mixed-variability system from industry [24]. In this type of system, variability is realized in multiple artifact types and with several variability techniques, for example at compile time using custom configuration tools or preprocessors, or during runtime via configuration files.

Rubin and Chechik present an algorithm for n-way merging of model variants which could be employed for combining the models of related projects into a product line [32]. Their operators *compare* and *compose* perform similar tasks as our extraction and composition. However, their focus is on merging variants rather than extracting traces from them as we do. Nonetheless, we should point out that our traces are in fact merged when creating (i.e., composing) products as explained in our evaluation.

Koschke et al. aim to reconstruct the module view of product variants and establish a mapping of code entities to architecture entities, with the goal of consolidating software product variants into software product lines by inferring the software product line architecture [22]. For this they adapt the reflexion method by applying it incrementally to a set of variants taking advantage of commonalities in their code, for which they use clone-detection and function similarity measures. In contrast to our work, they compute the mappings entirely based on source code and do not consider features (or feature interactions). Also, while we aim to keep our approach generic and applicable to different types of artifacts, we believe that, for the case of source code, our work could also benefit from clone-detection and function similarity metrics.

Rubin et al. propose a framework for managing product variants that are the result of clone-and-own practices [34]. They outline a series of operators and how they were applied in three industrial case studies. These operators serve to provide a more formal footing to describe the set of processes and activities that were carried out to manage the software variants in the different scenarios encountered in the case studies. We believe that our variability extraction techniques can provide the functionality of some of these operators, and we therefore plan to apply our techniques to such scenarios.

Xue et al. use diffing algorithms to identify the common and variable parts of product variants, which are subsequently partitioned using Formal Concept Analysis [38]. To these partitions, Information Retrieval algorithms are applied to identify the code units specific to a feature. In contrast to our work, they do not explicitly distinguish code of single features from code of feature interactions. However, we will explore how to leverage advanced diffing techniques employed in this work for detecting a wider spectrum of software artifact changes.

Rubin et al. survey feature location techniques for mapping features to their implementing software artifacts [33]. The extraction process in our work can also be categorized as a feature location technique, only that we also consider additional problems like feature interactions instead of just single features and also the order of artifacts instead of just their presence or absence. Another feature location survey exists by Dit et al. [12]. However, the approaches they survey do not identify feature interactions and dependencies as our work does.

Other traceability and information mining algorithms are presented by Ali et al. in [1] or by Kagdi et al. in [19] who use information retrieval techniques in combination with information mined from software repositories to locate features in the source code.

Chen et al. [9] present a way of displaying traceability links which could be used to visualize the traceability information extracted by our approach.

Nguyen et al. present JSync [30], a tool for managing clones in software systems. Techniques like these could be useful for us when performing the extraction on legacy product variants whose implementations have diverged significantly over time and feature implementations have become inconsistent across different product variants.

Laguna and Crespo performed a systematic mapping study on software product line evolution and made an assessment of the maturity level of the techniques by evaluating how suitable they are for industrial application, that is, if they have available methodology and tool support, and if they have been applied to relevant case studies [23]. They found that even though there is incipient work to address both challenges, the current methodological and tooling support is rather fragmented. Their work does corroborate the crucial need to develop an integrated framework, with robust methodological underpinnings and with adequate tool support, for which our work can be the foundation as variability information is crucial during any form of software product line evolution. Assuncao and Vergilio conducted another mapping study on feature location for the migration of software product lines [6]. The trace extraction we describe falls also under this category.

Martinez et al. present a generic and extensible approach for adopting software product lines from sets of product variants [29]. Similarly to our work, they also perform feature location and constraints discovery; however, they only consider single features and not feature interactions and instead of our rules they use another heuristic which is also based on commonalities and differences in product variants.

## Conclusions

In this work, we presented an approach for extracting variability information from sets of product variants. We extract traces from modules, a concept more flexible than simple features or requirements, to their implementing artifacts. We express traces in several degrees of certainty: where modules *at least* trace, where they can *at most* trace, and where they *certainly not* trace. Finally, we also compute dependencies between traces that can be used as a form of variability model. The evaluation using six case studies of various sizes and domains has shown promising results. From this information, all the input variants were correctly recomposed, and the dependencies between traces were consistent with the respective feature models.

## Future work

Our approach captures exactly the variability present in the used input product variants. However, if the variants have been maintained inconsistently (e.g., bug fixes applied to only some of the variants) and therefore have diverged from each other, also all the inconsistencies are captured. This may have a negative impact when using the traces to generate new, previously unknown, product variants that were not used as input. This is a result of *overfitting* [35] the extracted information to the input product variants. We plan on addressing this issue with configuration options expressing the desired degree of *fitting* of the extracted information to the used input product variants.

Also, looking at the extracted traces for ModelAnalyzer lead us to believe that often new features are the result of renaming of implementation artifacts (e.g., class or method names in the case of source code) or changing user interface strings. Using clone-detection techniques to account for this could enable our approach to extract more compact traces (e.g., instead of a whole new class implementing a new feature, it could be an already-existing class just with names changed).

We identified the computation and processing of modules as the part that consumes most of the runtime. Improving the performance of this aspect of our approach is a priority for our future work.

Another interesting avenue for further research lies in the exploration of the relation between code metrics expressing how modular a software system is, like for example cohesion or coupling, and the metrics computed by our approach, like the maximum order of modules or the distinguishability.

We are currently searching for more case studies, from other domains, with larger number of features and artifacts, ideally also containing other artifacts than source code such as UML or SysML diagrams.

## Footnotes

1
http://docs.oracle.com/javase/7/docs/jdk/api/javac/tree/.

2
http://www.eclipse.org/jdt/.
